# Pregnancy incidence and outcome before and after cervical intraepithelial neoplasia: a retrospective cohort study

**DOI:** 10.1002/cam4.300

**Published:** 2014-08-21

**Authors:** Ilkka Kalliala, Ahti Anttila, Pekka Nieminen, Mervi Halttunen, Tadeusz Dyba

**Affiliations:** 1 Department of Obstetrics and Gynaecology, Kätilöopisto Hospital, Helsinki University Central HospitalSofianlehdonkatu 5 A, 00029 HUS, Helsinki, Finland; 2 Mass Screening Registry, Finnish Cancer RegistryHelsinki, Finland; 3 Department of Obstetrics and Gynaecology, Helsinki University Central HospitalHelsinki, Finland; 4 Finnish Cancer RegistryHelsinki, Finland

**Keywords:** Cervical intraepithelial neoplasia, CIN, conization, fecundity, pregnancy outcome

## Abstract

We performed a retrospective cohort study of 3530 women treated for cervical intraepithelial neoplasia (CIN) in Helsinki University Central Hospital, Finland, to investigate whether CIN treatment itself affects pregnancy incidence and outcome. We estimated the incidence of live births, miscarriages, extrauterine pregnancies, molar pregnancies, and termination of pregnancies (TOPs) before and after CIN treatment using nationwide registers. Women were followed up until death, emigration, sterilization, or the end of 2004. The comparison of incidence of pregnancy outcomes before and after the treatment was estimated by calculating hazard ratios (HRs) with conditional Poisson regression. After 76,162 woman-years of follow-up, the incidence of any pregnancy remained constant over CIN-treatment, HR 1.02 and 95% confidence interval (CI) 0.97–1.08, but the incidence of the first pregnancy was significantly elevated after treatment, HR 1.13, and 95% CI 1.03–1.23. The incidence of live births was significantly elevated after treatment, HR 1.08 and 95% CI 1.01–1.15. Incidence of miscarriages, TOPs, extrauterine pregnancies, and molar pregnancies was not elevated. TOPs was significantly increased in the first pregnancy, HR 1.40, 95% CI 1.15–1.72 and after treatment by the loop electrosurgical excision procedure (LEEP), HR 1.36, 95% CI 1.15–1.60. CIN treatment did not reduce pregnancy incidence and women had more live births after than before CIN treatment. TOPs was more common in the first pregnancy or after treatment by LEEP. We encourage research on the psychosocial consequences of CIN treatment also in other countries and settings.

## Introduction

Evidence linking cervical intraepithelial neoplasia (CIN) treatments, like cervical conization, to later increase in preterm deliveries, low birthweight, and perinatal mortality is extensive [1–3]. Effects of the cervical conization itself on the incidence of pregnancies, live births, and other types of pregnancies are less clear. The largest study so far showed that the incidence of pregnancy or live birth was actually higher among the CIN-treated women compared to the age-matched reference population, and also the risk of induced abortion and extrauterine pregnancy was higher in CIN patients than the reference cohort [4].

Women with CIN differ from the general population in several significant ways: they smoke more, have more sexually transmitted infections, are more often multiparous, have more sexual partners during their lives, and they are younger at sexual debut [5]—all these factors are capable of affecting fertility as well. Therefore, comparing the treated to healthy might only reflect differences in group-specific fertility patterns and hence dilute or even mask the direction and magnitude of the true effect of the intervention, CIN treatment, on the studied outcomes.

Our objective was to examine the effects of CIN treatment itself upon incidence of live births and other pregnancy outcomes—miscarriages, abortions, extrauterine pregnancies, and molar pregnancies—by comparing the CIN patients' pregnancy outcomes before and after CIN treatment with those within a cohort of CIN-treated women. The previous analysis [4] could not adequately take into account the above factors; there could have been residual confounding left in the results—they may not reflect merely the effect of CIN treatment itself on the outcomes studied, but rather the differences between women with and without CIN. The current study offers a more appropriate approach to study the effect of CIN treatment.

## Material and Methods

To study the possible effect of CIN treatment on pregnancy outcome, we compared the incidence of different types of pregnancies before and after CIN treatment within a cohort of CIN-treated women. The initial study group comprised 7253 women treated for CIN with cold knife conization (CKC), cryocoagulation (cryo), laser conization or ablation, or with a loop electrosurgical excision procedure (LEEP), between 1974 and 2001 at Helsinki University Central Hospital, Finland, a reference center for colposcopy in the district.

In our data, median age at treatment was lower than at the first pregnancy, so women were more likely to be pregnant after than before the CIN treatment. Presenting results of a cohort where treated women serve as their own controls, adjusting only for age at treatment and age at endpoint does not sufficiently control for age, therefore we fitted all models for a matched reference cohort as well. For each woman treated, five individually age- and municipality-matched controls were selected from the Finnish Population Register, resulting in 36,265 women in the initial reference cohort.

Data covering all pregnancy outcomes were available only from the beginning of 1974. Hence, from the initial study population we included in the study only women turning 16 at the onset of nationwide registers or younger, 1974, that is, women born 1958 or later. After this exclusion, the final study population included 3530 women treated for CIN and the reference cohort was reduced to 17,451 women.

Birth dates of their live-born children (date of live singleton birth) and possible dates of emigration or death were retrieved from the population registry. Precise dates of all other pregnancy outcomes (miscarriage, extrauterine pregnancy, molar pregnancy, and termination of pregnancy [TOP]) and the date of possible sterilization were gathered from the THL's (National Institute for Health and Welfare) Care Registers for Social Welfare and Health, who have been shown to have high completeness and validity [6].

A live birth here means delivery of a live-born baby with over 24 weeks of gestation; miscarriages occurred before 24 gestational weeks; extrauterine pregnancy is pregnancy outside the uterus; molar pregnancy means benign gestational trophoblastic neoplasia; and TOP means a medically performed abortion, either by medication or by dilatation and curettage. Pregnancy incidence denotes incidence of all endpoints: live births, TOPs, extrauterine pregnancies, miscarriages, and molar pregnancies.

The incidence of the outcomes studied was calculated over consecutive follow-up periods for each woman in both cohorts to account for the changing values of explanatory variables over the whole follow-up time. We calculated the conception time of each pregnancy by median estimates of the duration of pregnancy for each outcome and were thus able to determine whether a pregnancy had started before or after the CIN treatment. (1) A live birth: 280 days before the actual birth. (2) Miscarriage or TOP: 63 days before. (3) Extrauterine pregnancy: 56 days. (4) Molar pregnancy: 77 days. All women were followed up until sterilization, turning 50, emigration, death, or at the end of 2004. The median age at treatment was 26 and age at first pregnancy among the 2637 treated women who became pregnant at least once was 24.

### Statistical analysis

We performed an internal analysis within the cohort of CIN-treated women and the reference cohort independently. Hazard ratios (HRs) were calculated by comparing the adjusted incidence of each endpoint after versus before the treatment of CIN using conditional Poisson regression [7]. The adjusted model-based HR—the final results presented in Tables 2 and 3—then took the form of the ratio between the model-based HRs for the treated and the model-based hazard rate for the reference group. In this way, the final results for the treated women could be adjusted for the general pattern of fertility in the general population represented in our study by the reference cohort. Comparisons between risks were reported as HRs with 95% confidence intervals (CIs). Statistical significance of the analyzed variables was obtained by comparing appropriate hierarchical models. The CIs were calculated by the delta method [8].

All models were adjusted for the number of pregnancies (0, 1, 2, 3+) and children (0, 1, 2, 3+), age at treatment and age at the beginning of each pregnancy, municipality, grade of CIN, treatment method, calendar year, and whether the type of pregnancy in question had already occurred before the treatment.

In addition to the overall incidence of each pregnancy outcome, separate similar models were fitted specific to the number of pregnancies or live births before the index pregnancy (no pregnancies before, pregnancy or pregnancies but no live births before, one child before, two children before, or three or more children before the index pregnancy), specific to the grade of CIN (CIN 1–3), and to the current method of treatment, LEEP. All statistical analyses were performed with STATA software (StataCorp 2011, Stata Statistical Software: release 12.1, College Station, TX).

The research protocol of this study has been approved by the Ethics Committee Section for Obstetrics and Gynaecology in the Helsinki-Uusimaa Hospital District (Admission date: 15 August 2003, 150/E8/03).

## Results

Among the 3530 CIN-treated women with 76,162 woman-years of follow-up, we observed altogether 6535 pregnancies of which 4615 were live births, 1545 TOPs, 208 miscarriages, 97 extrauterine pregnancies, and 70 molar pregnancies (Table 1).

**Table 1:** Numbers of women, woman-years, and endpoints.

	*N*	Woman-years	Pregnancies	Live births	Termination of pregnancy	Miscarriages	Extrauterine pregnancies	Molar pregnancies
Overall treated	3530	76,162	6535	4615	1545	208	97	70
Before		42,032	2827	1730	927	94	45	31
After		34,130	3708	2885	618	114	52	39
Overall reference	17,451	376,594	28,499	21,926	5193	851	266	263
Before		205,810	12,504	8560	3307	379	146	112
After		170,784	15,995	13,366	1886	472	120	151
CIN 1 total	1,180	24,141	2002	1419	465	80	22	16
Before		13,406	809	482	269	39	12	7
After		10,735	1193	937	196	41	10	9
CIN 2 total	1720	38,335	3367	2367	797	104	58	41
Before		19,425	1301	771	456	39	19	16
After		18,910	2066	1596	341	65	39	25
CIN 3 total	630	13,686	1166	829	283	24	17	13
Before		9201	717	477	202	16	14	8
After		4485	449	352	81	8	3	5
LEEP total	2317	46,220	3588	2569	851	92	38	38
Before		32,427	2168	1422	613	71	35	27
After		13,793	1420	1147	238	21	3	11

Overall for treated and reference cohort separately. For the treated only according to the grade of CIN, and for those treated with LEEP. All numbers total, before, and after the treatment in all categories. CIN, cervical intraepithelial neoplasia; LEEP, loop electrosurgical excision procedure.

The incidence of any pregnancy was similar after and before the CIN treatment, HR 1.02 and 95% CI 0.97–1.08, but the incidence of the first-ever pregnancy was significantly elevated after treatment, HR 1.13 and 95% CI 1.03–1.23 (Table 2). The incidence of live births was significantly higher after overall treatment, HR 1.08 and 95% CI 1.01–1.15, in the first-ever pregnancy, HR 1.16 and 95% CI 1.04–1.28, and in pregnancies after one or two children (Table 2). Overall incidences of TOPs, miscarriages, molar pregnancies, and extrauterine pregnancies were not statistically significantly increased after CIN treatment (Table 2). Overall incidence of TOPs was slightly but not significantly elevated, HR 1.07 and 95% CI 0.95–1.20, and reached statistical significance when the first-ever pregnancy was TOP, HR 1.40 and 95% CI 1.15–1.72 (Table 2). Incidence of extrauterine pregnancies was constantly elevated after treatment, regardless of pregnancy history, but the difference was statistically significant only in pregnancies that began after one child HR 3.50 and 95% CI 1.36–9.03 (Table 2).

**Table 2:** Pregnancy outcome.

	Pregnancies	Live births	Termination of pregnancy*	Miscarriages*	Extrauterine pregnancies*	Molar pregnancies*
Overall	1.02 (0.97–1.08)	1.08 (1.01–1.15)	1.07 (0.95–1.20)	0.93 (0.68–1.27)	1.19 (0.73–1.95)	0.93 (0.54–1.62)
First ever pregnancy	1.13 (1.03–1.23)	1.16 (1.04–1.28)	1.40 (1.15–1.72)	1.21 (0.72–2.04)	1.37 (0.61–3.08)	0.99 (0.39–2.51)
No live births before	0.95 (0.82–1.10)	0.93 (0.84–1.04)	1.06 (0.77–1.45)	0.94 (0.41–2.19)	1.28 (0.43–3.80)	0.52 (0.12–2.14)
1 live birth	1.14 (1.03–1.26)	1.20 (1.07–1.35)	1.09 (0.84–1.43)	0.95 (0.56–1.61)	3.50 (1.36–9.03)	0.96 (0.39–2.36)
2 live births	1.12 (0.93–1.34)	1.45 (1.15–1.82)	0.82 (0.59–1.14)	0.83 (0.32–2.16)	0.46 (0.12–1.81)	1.73 (0.30–10.0)
3+ live births	0.77 (0.55–1.10)	0.87 (0.56–1.35)	0.59 (0.32–1.23)	0.11 (0.01–0.75)	NA	NA

Hazard ratios of all endpoints, after versus before treatment of CIN. All results are based on conditional Poisson regression models [6] by comparing the incidence of outcome in question before versus after the CIN treatment. All models are adjusted for the method of CIN treatment, grade of histology, age at treatment, age at endpoint, place of residence, calendar year, and age- and municipality-matched reference population. The overall results are furthermore adjusted for pregnancy history. Other results are retrieved from stratified models according to pregnancy history: women with no pregnancies before the index pregnancy (the first-ever pregnancy); women with pregnancy or pregnancies but no live births before the index pregnancy (no live births before); and women with 1, 2, or 3 or more live births before the index pregnancy. NA, not available due to toofew observations; CIN, cervical intraepithelial neoplasia; LEEP, loop electrosurgical excision procedure.

* Adjusted also for whether the endpoint in question had already occurred at least once.

We did not observe significant differences between the grades of CIN in any of the outcomes studied (Table 3). Results of those women treated with LEEP did not significantly differ from those of the whole study population, although the incidence of TOPs was statistically significantly and higher after than before the treatment, HR 1.36 and 95% CI 1.15–1.60 (Table 3).

**Table 3:** Overall pregnancy outcomes according to grade of CIN and for LEEP separately.

	Pregnancies	Live births	Termination of pregnancy*	Miscarriages*	Extrauterine pregnancies*	Molar pregnancies*
CIN 1	1.11 (1.02–1.22)	1.20 (1.07–1.35)	1.21 (1.00–1.47)	0.92 (0.58–1.45)	1.11 (0.46–2.67)	1.04 (0.38–2.89)
CIN 2	0.99 (0.92–1.06)	1.05 (0.96–1.15)	1.04 (0.90–1.21)	0.95 (0.62–1.45)	1.54 (0.84–2.82)	0.76 (0.39–1.50)
CIN 3	0.97 (0.86–1.10)	0.96 (0.83–1.10)	1.30 (1.00–1.69)	0.95 (0.40–2.24)	0.84 (0.23–3.03)	1.40 (0.44–4.44)
LEEP	1.10 (1.03–1.22)	1.12 (1.03–1.22)	1.36 (1.15–1.60)	0.74 (0.44–1.24)	0.73 (0.21–2.50)	2.52 (0.93–4.41)

All results based on conditional Poisson regression models [6] by comparing the incidence of outcome in question before versus after CIN treatment. Results for CIN grades 1–3 are adjusted for pregnancy history, for the method of CIN treatment, age at treatment, age at endpoint, place of residence, calendar year, and age- and municipality-matched reference population. Results for LEEP are adjusted for pregnancy history, the grade of histology, age at treatment, age at endpoint, place of residence, calendar year, and age- and municipality-matched reference population; CIN, cervical intraepithelial neoplasia.

* Adjusted also for whether the endpoint in question had already occurred at least once.

## Discussion

Among over 3500 CIN-treated women with 76,000 woman-years of follow-up, we observed a significant increase in the incidence of live births after CIN treatment. Pregnancy incidence remained stable through the CIN treatment, but significantly more women became pregnant at least once after treatment.

In the current study, all comparisons before and after pregnancy were made within the cohort of treated women and reference cohort and all models are adjusted for all possible confounding factors retrievable from the registers used (parity, age, method of treatment, grade of CIN, place of residence, previous incidence of the outcome studied, and time of treatment). This setting allows for drawing broader conclusions about the direct effect of CIN treatment itself on later fertility than in a setting where the comparisons are made between the treated and a healthy reference group [4].

The number of miscarriages and extrauterine pregnancies in the data is small, and many results concerning these endpoints lack statistical power. This is most likely due to the fact that mainly those women who were taken as in-patients appeared in nationwide registers during the study period. This remained the same throughout the study period and hence does not systematically bias the results. As women treated for CIN were collected from the hospital register, some women might have been treated for CIN before the first treatment in our data or might have been treated again later. This phenomenon would rather dilute than exaggerate the results observed in this setting and as these events have been rare, these cannot therefore be considered to seriously bias the conclusions.

Pregnancy incidence and especially live birth incidence increased after CIN treatment. All results were adjusted for age-specific fertility patterns of general population, so a natural increase in reproductive activity with advancing age (median age at treatment was 26) does not explain this observation. Most likely the surgical procedure itself does not directly promote reproduction either. We know that CIN treatment causes anxiety and distress [5, 9]. Even though the psychosocial consequences of CIN treatment was not studied here, we cannot rule out that a psychosocial effect of CIN treatment might increase rather than decrease the will to acquire children.

In our previous study, the incidence of different pregnancy types was compared between CIN-treated women and the healthy reference cohort. Incidences of TOPs and extrauterine pregnancy were significantly elevated among the treated women [4]. In the current study, overall TOP incidence was not increased. However, among women treated with LEEP (66% of the study population) and in the first-ever pregnancy, TOPs were significantly more common after treatment. As LEEP is the dominating treatment method in the most recent periods (used in the current hospital settings since 1991), these two findings could correlate to each other. The CIN treatment may hence cause a small but significant increase in TOP incidence. As the majority, over 95%, of TOPs in Finland, is made due to social reasons [6], the psychological effects of CIN treatment might play a small role in the observed increase in TOPs incidence after treatment. However, supporting direct evidence was beyond the scope of our study setting.

Incidence of extrauterine pregnancies was slightly increased after treatment, but due to the small number of events in the data all but one result lacked statistical significance, and strong conclusions cannot therefore in our opinion be made.

The overall incidence of miscarriages was not increased, and a slight but not statistically significant increase was observed only among women with no previous pregnancy. If the CIN treatment was subject to miscarriages, the incidence should be elevated regardless of pregnancy history.

Cervical conization effectively prevents cervical cancer [10]. A significant number of these procedures are made for women during their fertile years. Even though vast evidence links conization to preterm delivery, our findings are reassuring for physicians: the pregnancy incidence after CIN treatment did not decrease and adjusted livebirth incidence was higher after than before CIN treatment. As the results are based on a large data set with a nationwide follow-up of women treated in a public hospital, we consider our findings' generalisability as satisfactory for our country. We did observe a significant increase in TOP incidence after LEEP and in the first pregnancy as well, and increased reproductive activity after the treatment. In light of the findings of the current study, more research about the psychosocial consequences of CIN treatment is strongly encouraged in addition to countries and settings.
